# Spontaneous virologic suppression in HIV controllers is independent of delayed-type hypersensitivity test responsiveness

**DOI:** 10.1186/1742-6405-9-10

**Published:** 2012-04-02

**Authors:** Jason F Okulicz, Greg A Grandits, Matthew J Dolan, Vincent C Marconi, Glenn Wortmann, Michael L Landrum

**Affiliations:** 1 Infectious Disease Clinical Research Program, Uniformed Services University of the Health Sciences, Bethesda, MD, USA; 2 San Antonio Military Medical Center, Infectious Disease Service, 3551 Roger Brooke Drive, Fort Sam Houston, TX 78234, USA; 3 Division of Biostatistics, University of Minnesota, Minneapolis, MN, USA; 4 Henry M. Jackson Foundation, Lackland AFB, TX, USA; 5 Emory University School of Medicine, Atlanta, GA, USA; 6 Infectious Disease Service, Walter Reed National Military Medical Center, Bethesda, MD, USA

**Keywords:** HIV, Elite controllers, HIV controllers, Delayed-type hypersensitivity test, HAART

## Abstract

**Background:**

Delayed-type hypersensitivity (DTH) testing, an in vivo assessment of cell-mediated immunity, is a predictor of HIV disease progression beyond CD4 cell count. We investigated whether preserved DTH responsiveness was characteristic of HIV controllers compared to non-controllers and individuals on suppressive HAART.

**Findings:**

DTH testing consisted of ≥ 3 recall antigens applied approximately every 6 months. DTH responses were classified by the number of positive skin tests: anergic (0), partial anergic (1), or non-anergic (≥ 2). HIV controllers were compared to treatment naïve non-controllers (n = 3822) and a subgroup of non-controllers with VL < 400 copies/mL on their initial HAART regimen (n = 491). The proportion of non-anergic results at first DTH testing was similar for HIV controllers compared to non-controllers (81.9% vs. 77.6%; P = 0.22), but tended to be greater in HIV controllers compared to the HAART subgroup (81.9% vs. 74.5%; P = 0.07). Complete anergy was observed in 14 (10.1%) HIV controllers with CD4 counts ≥ 400 cells/uL. For longitudinal testing, the average percentage of non-anergic DTH determinations per participant was higher in HIV controllers compared to non-controllers (81.2 ± 31.9% vs. 70.7 ± 36.8%; P = 0.0002), however this difference was eliminated with stratification by CD4 count: 200-399 (83.4 ± 35.6% vs. 71.9 ± 40.9%; P = 0.15) and > 400 cells/uL (81.2 ± 31.5% vs. 80.4 ± 32.7%; P = 0.76).

**Conclusions:**

Spontaneous virologic control was not associated with DTH responsiveness, and several HIV controllers were anergic despite having elevated CD4 counts. These findings suggest that cellular immunity assessed by DTH is not a principal factor contributing to spontaneous virologic suppression in HIV controllers.

## Introduction

Delayed-type hypersensitivity (DTH) testing can be used as an in vivo assessment of cell-mediated immunity (CMI). Compared to HIV-seronegative individuals, patients with HIV typically have less favorable DTH responses, particularly in the setting of low CD4 cell counts where anergy is common [1,2]. Among HIV-infected persons on highly active antiretroviral therapy (HAART), DTH responsiveness has been shown to be both a predictor of treatment outcomes and a marker for improved CMI [3-5].

Elite and viremic controllers, collectively termed HIV controllers, are characterized by the ability to spontaneously control plasma HIV viral load (VL) for prolonged periods without HAART [6,7]. HIV controllers typically have several characteristics similar to HAART-suppressed individuals, including elevated CD4 counts and reduced risk of AIDS and death [6]. We investigated whether DTH responsiveness was greater in HIV controllers compared to non-controllers and HAART suppressors in the U.S. Military HIV Natural History Study (NHS).

## Methods

The NHS is a prospective observational cohort of over 5300 military members, dependents, and beneficiaries with HIV-1 infection followed in the military healthcare system since 1986 [8]. Participants providing informed consent in this IRB-approved study are evaluated approximately every 6 months at selected US military treatment facilities.

HIV controllers are composed of 2 mutually exclusive groups termed elite and viremic controllers as defined previously [6]. Elite controllers were defined as having ≥ 3 plasma VLs below the limit of detection spanning ≥ 12 months without HAART. Viremic controllers were defined as having ≥ 3 VLs ≤ 2000 copies/mL over a period of ≥ 12 months without HAART. Non-controllers were cohort participants not meeting HIV controller definitions. A subset of non-controllers, termed HAART suppressors, was defined as those achieving a VL < 400 copies/mL within the first 6 months of their initial HAART regimen.

DTH testing was performed according to standardized protocols as previously described [1,2,5,9]. A total of 0.1 mL of each antigen was applied to the forearm intradermally according to the Mantoux method and a positive test was defined as ≥ 5 mm of induration after 48 h. The most recent antigens and concentrations included tetanus toxoid (Lederle 1.6 Lf/mL; 1:100 dilution), mumps (Connaught, 40 CFU/mL), trichophyton (Holister-Stier, 1:500 dilution), and candida (Walter Reed Army Institute of Research, 200 PNU/mL). Participants received a panel of 3-4 antigens, with the majority receiving 3 antigens as trichophyton was removed from the market in 1996. DTH responses were categorized by the number of positive skin tests: anergic (0), partial anergic (1), or non-anergic (≥ 2) as previously described [1].

First DTH response refers to the initial DTH determination performed during the period of spontaneous virologic control for HIV controllers and the first available DTH test for non-controllers. First DTH response for HAART suppressors was defined as the DTH determination after 2 years of HAART. The proportion of all DTH determinations with non-anergic results was also studied for those with ≥ 2 DTH testing episodes.

Statistical comparisons were made between HIV controllers and both non-controllers and HAART suppressors. Demographic and HIV-1 characteristics were compared using t-tests for continuous variables and chi-square tests for categorical variables. First DTH test results were compared using chi-square tests. Longitudinal DTH measures for each subject were summarized as the percentage of test results that were non-anergic and between-group comparisons were made using weighted analysis of variance. Weights were a function of the number of measurements available per subject and the within and between subject components of variance of DTH results. Analyses are presented overall and by CD4 count at the time of DTH testing. Analysis was also done adjusting for CD4 count as a continuous variable.

## Results

DTH testing was performed in 33 elite and 116 viremic controllers (Table 1). There were 3822 non-controllers for comparison, of which 491 also met criteria for the HAART suppressor subgroup. Both HIV controllers and non-controllers were predominantly male and approximately 29 years at HIV-1 diagnosis, but HIV controllers had a higher proportion of African Americans (P < 0.01). HIV controllers had both a later calendar year of diagnosis (1995 ± 6 years) and first DTH test (1997 ± 5) compared to non-controllers (1991 ± 5 and 1992 ± 5, respectively; P < 0.01 for both). HIV controllers had a lower log_10 _VL at diagnosis (3.0 ± 0.8) compared to non-controllers (4.3 ± 0.8; P < 0.01) and the HAART suppressor subgroup (4.4 ± 0.8; P < 0.01). Mean CD4 counts were also higher at HIV-1 diagnosis for HIV controllers (723 ± 234 cells/uL) compared to non-controllers (539 ± 275 cells/uL; P < 0.01) and the HAART suppressor subgroup (505 ± 243 cells/uL; P < 0.01). The number of DTH testing episodes for HIV controllers and non-controllers was similar (5.5 ± 4.8 vs. 5.5 ± 4.7; P = 0.84).

**Table 1:** Characteristics of HIV controllers, non-controllers, and HAART suppressors

Characteristic	Elite Controllers	Viremic Controllers	HIV Controllers	Non- Controllers	P-value HIV Controllers vs. Non- controllers	HAART Suppressors	P-value HIV Controllers vs. HAART Suppressors
Number of Participants, n	33	116	149	3822	-	491	-

Age at HIV Diagnosis (years)	29.3 ± 7.4	28.8 ± 6.6	28.9 ± 6.8	29.4 ± 7.8	0.45	31.1 ± 7.9	0.002

Gender, Male	30 (90.9)	102 (87.9)	132 (88.6)	3512 (91.9)	0.15	450 (91.6)	0.25

Race/Ethnicity							

European American	12 (36.4)	41 (35.3)	53 (35.6)	1725 (45.2)	0.021	244 (49.7)	0.002

African American	21 (63.6)	64 (55.2)	85 (57.0)	1668 (43.7)	0.001	192 (39.1)	< 0.001

Hispanic	0 (0.0)	6 (5.2)	6 (4.0)	300 (7.9)	0.09	38 (7.7)	0.12

Other	0 (0.0)	5 (4.3)	5 (3.4)	127 (3.4)	0.98	17 (3.5)	0.95

Year of HIV Diagnosis	1993 ± 6	1995 ± 6	1995 ± 6	1991 ± 5	< 0.001	1995 ± 5	0.90

Mean CD4^+ ^Count at HIV Diagnosis (cells/uL)	689.7 ± 241.6	731.9 ± 232.6	723.4 ± 234.0	539.2 ± 275.1	< 0.001	504.7 ± 242.6	< 0.001

Mean Viral Load at HIV Diagnosis (log_10 _copies/mL)	2.7 ± 0.8	3.0 ± 0.8	3.0 ± 0.8	4.3 ± 0.8	< 0.001	4.4 ± 0.8	< 0.001

Year of First DTH Test^a^	1997 ± 4	1997 ± 5	1997 ± 5	1992 ± 5	< 0.001	2000 ± 2	< 0.001

Mean Number of DTH Testing Episodes	4.9 ± 4.2	5.7 ± 5.0	5.5 ± 4.8	5.5 ± 4.7	0.84	N/A	N/A

**NOTE**. HIV controllers, combined group of elite and viremic controllers; HAART suppressors, subgroup of non-controllers on suppressive HAART; all values are number, percent or SD unless otherwise specified

^a ^First DTH for HAART subgroup defined as first DTH test 2 years after start of initial HAART regimen

A non-anergic DTH determination (response to ≥ 2 antigens) was not universally observed for elite (72.7%) and viremic controllers (84.5%) at first DTH evaluation. In the combined group of HIV controllers, the percentage of non-anergic DTH results was somewhat higher than non-controllers (81.9% versus 77.6%) but the difference was not significant (P = 0.22) (Table 2). When stratified by CD4 count at DTH testing, the proportion with non-anergic DTH tests was no different for HIV controllers compared to non-controllers, with 70% vs. 74.2% non-anergic at CD4 200-399 cells/uL (P = 0.76) and 82.6% vs. 84.7% at CD4 ≥ 400 cells/uL (P = 0.52), respectively. In a model adjusted for CD4 count there was no significant differences between groups. Surprisingly, 4 (12.9%) elite and 10 (9.3%) viremic controllers were completely anergic despite having CD4 counts ≥ 400 cells/uL.

**Table 2:** Delayed-type hypersensitivity test results for HIV controllers compared to non-controllers and HAART suppressors

Characteristic	Elite Controllers	Viremic Controllers	HIV Controllers	Non- Controllers	P-value HIV Controllers vs. Non- Controllers	HAART Suppressors	P-value HIV Controllers vs. HAART Suppressors
Number of Participants, n	33	116	149	3822	-	491	-

DTH Test Results, All Participants							

First DTH Test Results^a^							

Non-anergic	24 (72.7)	98 (84.5)	122 (81.9)	2965	0.22	366 (74.5)	0.07

				(77.6)			

Partial Anergy	5 (15.2)	7 (6.0)	12 (8.1)	475 (12.4)	-	62 (12.6)	-

Complete Anergy	4 (12.1)	11 (9.5)	15 (10.1)	382 (10.0)	-	63 (12.8)	-

Average Percent of DTH Test Events Non-anergic^b^	78.0 ± 31.3 (n = 33)	82.0 ± 32.2 (n = 116)	81.2 ± 31.9 (n = 149)	70.7 ± 36.8 (n = 3822)	0.0002	-	-

Participants with CD4 200-399 cells/uL at DTH Testing							

First DTH Test Results							

Non-anergic	1 (50.0)	6 (75.0)	7 (70.0)	670 (74.2)	0.76	52 (66.7)	0.83

Partial Anergy	1 (50.0)	1 (12.5)	2 (20.0)	138 (15.3)	-	14 (17.9)	-

Complete Anergy	0 (0)	1 (12.5)	1 (10.0)	95 (10.5)	-	12 (15.4)	-

Average Percent of DTH Test Events Non-anergic	65.1 ± 57.7 (n = 3)	85.8 ± 33.4 (n = 27)	83.4 ± 35.6 (n = 30)	71.9 ± 40.9 (n = 2116)	0.15	-	-

Participants with CD4 ≥ 400 cells/uL at DTH Testing							

First DTH Testing Results							

Non-anergic	23 (74.2)	91 (85.0)	114 (82.6)	1910 (84.7)	0.52	306 (76.5)	0.14

Partial Anergy	4 (12.9)	6 (5.6)	10 (7.2)	222 (9.8)	-	47 (11.8)	-

Complete Anergy	4 (12.9)	10 (9.3)	14 (10.1)	124 (5.5)	-	47 (11.8)	-

Average Percent of DTH Test Events Non-anergic	78.2 ± 31.5 (n = 32)	82.0 ± 32.8 (n = 109)	81.2 ± 31.5 (n = 141)	80.4 ± 32.7 (n = 2705)	0.76	-	-

**NOTE**. HIV controllers, combined group of elite and viremic controllers; HAART suppressors, subgroup of non-controllers on suppressive HAART; all values are number, percent or SD

^a ^Non-anergic, ≥ 2 tests positive; partial anergy, 1 test positive; complete anergy, zero positive tests

^b ^Weighted average of each participant's individual average. Weights based on number of measurements per subject and correlation of repeated DTH measurements

The average percent of all DTH determinations with a non-anergic result was high for both elite (78.0 ± 31.3%) and viremic (82.0 ± 32.2%) controllers. Compared to non-controllers, the average percent with a non-anergic result was higher in HIV controllers (81.2 ± 31.9% vs. 70.7 ± 36.8%; P < 0.01). However, after adjusting for CD4 count there was no significant difference between groups (P = 0.98); among those with CD4 ≥ 400 cells/uL, the average percent non-anergic was nearly identical (81.2 vs. 80.4%, P = 0.76).

Of the 491 HAART suppressors, the majority (70.1%) were prescribed protease inhibitor-based regimens and the mean CD4 count and log_10 _VL at HAART initiation was 411 ± 217 cells/uL and 4.2 ± 1.0 copies/mL, respectively. At the time of first DTH determination 2 years after starting HAART, the median increase in CD4 count was 226 cells/uL (IQR, 108-356). HIV controllers tended to have a greater proportion of non-anergic results compared to those on ≥ 24 months of HAART (81.9% vs. 74.5%; P = 0.07; Table 2), however this difference diminished when stratified by CD4 level (82.6% vs. 76.5%; P = 0.14 for CD4 ≥ 400 cells/uL).

## Discussion

HIV controllers, though defined by virologic criteria, are typically associated with elevated CD4 cell counts and improved clinical outcomes [6,7]. We determined that spontaneous virologic suppression in HIV controllers was independent of DTH responsiveness since nearly one-fifth of HIV controllers displayed partial or complete anergy at first DTH testing despite higher CD4 counts, and a similar proportion of non-anergic results were observed between HIV controllers and non-controllers when stratified by CD4 count.

A previous study [4] in the Air Force component of our HIV-infected population showed that 86% of participants with CD4 count > 400 cells/uL were non-anergic, similar to the 83% and 85% observed for HIV controllers and non-controllers, respectively in our study. Though HIV controllers typically have preserved DTH responses at higher CD4 cell counts, a proportion displayed anergy to recall antigens. Among elite controllers, 26% demonstrated anergy (4 partial and 4 complete) at first DTH testing despite having CD4 cell counts ≥ 400 cells/uL. This suggests that factors contributing to virologic control and DTH responsiveness do not completely overlap. We previously showed that a favorable CCL3L1-CCR5 genetic risk group (GRG) status, which is enriched in the HIV controller population, was associated with greater DTH responsiveness [5]. However, approximately 25% of HIV controllers did not have a favorable CCL3L1-CCR5 GRG status which suggests that elite and viremic controllers may represent a convergence of heterogeneous phenotypes with the common feature of spontaneous virologic control, and reinforces the concept of the presence of both viral load dependent and independent mechanisms of HIV-1 pathogenesis and host response.

In addition to reconstitution of CD4 cells, HAART impacts the immune system in other ways including the improvement of serologic response to vaccinations [10], reducing immune activation [11], and enhancing DTH responses [3,12]. In one study, suppression of plasma viremia was necessary for improved DTH responsiveness on HAART [13]. For HAART-naïve individuals, lower steady-state VL has also been associated with greater DTH responsiveness [5]. In comparison to participants with ≥ 2 years of suppressive HAART in our study, HIV controllers had a greater tendency for non-anergic DTH responses (81.9% vs. 74.5%; P = 0.07) and a similar pattern was observed when stratified by CD4 count. HAART suppressors also displayed less DTH responsiveness than the non-controllers group as a whole. This may be due to enrichment with participants who had declining immune function that led to the initiation of HAART.

Previous studies validated the use of multiple DTH antigen panels for studying various HIV outcomes [1,2,9]. Although a similar approach was used, a limitation of our study was that a comparison of individual antigens was not able to be performed. In addition, other virus-related factors that can influence HIV-1 disease progression, such as viral fitness and HIV clade, were not examined. Future studies investigating non-anergic versus anergic DTH responses, including higher resolution ex vivo assays of CMI to recall antigens and other immunologic studies, may provide additional insight into DTH responsiveness.

Although virologic control occurred by different mechanisms, both HIV controllers and HAART suppressors commonly displayed preserved DTH responses, especially at higher CD4 counts. A proportion of HIV controllers were anergic at DTH testing despite higher CD4 cell counts. Thus, HIV controller phenotypes appear to achieve virologic control by disparate mechanisms than those involving DTH responsiveness to recall antigens.

## Competing interests

The authors declare that they have no competing interests.

## Authors' contributions

All authors participated in the design of the study and manuscript preparation. GAG performed the statistical analysis. All authors read and approved the final manuscript.
